# Hepatoprotective effect of crude polysaccharides extracted from *Ganoderma lucidum* against carbon tetrachloride-induced liver injury in mice

**DOI:** 10.14202/vetworld.2019.1987-1991

**Published:** 2019-12-17

**Authors:** Raden Joko Kuncoroningrat Susilo, Dwi Winarni, Saikhu Akhmad Husen, Suhailah Hayaza, Hunsa Punnapayak, Sri Puji Astuti Wahyuningsih, Elma Sakinatus Sajidah, Win Darmanto

**Affiliations:** 1 Department of Biology, Faculty of Science and Technology, Universitas Airlangga, Surabaya 60115, Indonesia; 2 Plant Biomass Utilization Research Unit, Department of Botany, Faculty of Science, Chulalongkorn University, Bangkok 10330, Thailand

**Keywords:** carbon tetrachloride, crude polysaccharide extract, *Ganoderma lucidum*, hepatotoxicity

## Abstract

**Background and Aim:**

Natural products are currently widely used as alternative treatments for liver disease. The study aimed to determine the hepatoprotective effect of crude polysaccharides extracted from *Ganoderma lucidum* against liver injury induced by carbon tetrachloride (CCl_4_).

**Materials and Methods:**

Twenty-four male BALB/C mice were randomly divided into six groups. Serum and liver samples were taken on day 10 after *G. lucidum* administration. The levels of alanine aminotransferase (ALT), aspartate aminotransferase (AST), malondialdehyde (MDA), superoxide dismutase (SOD), and catalase (CAT) were measured using enzyme-linked immunosorbent assays, and the histology of the liver was evaluated using light microscopy.

**Results:**

*G. lucidum* extract significantly decreased the levels of ALT, AST, and MDA and significantly increased the levels of SOD and CAT. In the histological evaluation, the liver tissue of CCl_4_-treated mice exhibited hydropic degeneration, necrosis, and sinusoidal dilatation. *G. lucidum* extract administration improved this liver tissue histopathology.

**Conclusion:**

Crude polysaccharides extracted from *G. lucidum* showed a hepatoprotective effect, regenerating damaged liver tissue.

## Introduction

The liver is an important homeostatic organ and is involved in the process of detoxifying harmful drugs and chemicals. Given that, the incidence of jaundice and hepatitis has increased the development of hepatoprotective drugs from natural sources which is necessary. Liver damage may arise from excessive alcohol intake or exposure to toxins and environmental pollutants and can progress into severe liver diseases such as hepatitis, cirrhosis, and liver cancer [1,2]. Reactive oxygen species (ROS) cause cellular damage in the liver [3], and increased ROS can be caused by exposure to toxic substances such as carbon tetrachloride (CCl_4_) [4]. Therefore, CCl_4_ is often used to induce liver damage in model animals. CCl_4_-induced hepatotoxicity occurs through a metabolic process by cytochrome P450 which produces trichloromethyl radicals. These react with sulfhydryl groups, for example, reduced glutathione and thiol proteins, disrupting the physiological function of liver cells and ultimately inducing cell necrosis [5]. These radicals increase lipid metabolism, resulting in a decrease in the transport of these lipids and causing steatosis or fatty liver. The breakdown of these radicals forms reactive aldehyde, which increases membrane permeability and ultimately causes cell death. The dominance of pro-oxidants over antioxidants causes oxidative stress which can damage proteins, carbohydrates, nucleotides, and lipids [6]. Liver sections of CCl_4_-treated mice show hepatocyte necrosis, hemorrhage, vacuolar change, and hydropic degeneration [7]. In addition, liver sections of CCl_4_-treated rabbits show a total loss of hepatic architecture with fatty changes, sinusoidal congestion, and intense necrosis [8].

Antioxidants could potentially be used for the prevention and treatment of many diseases associated with ROS. Polysaccharides extracted from fungi, plants, bacteria, and algae are potential sources of antioxidants [9]. Natural products are currently widely used as alternative treatments for liver disease [10]. Polysaccharides are the active compounds of the *Ganoderma lucidum* fungus and can be extracted from the fruiting body and mycelium. The previous research has shown that polysaccharides have immunomodulatory, anticancer, and anti-aging effects and can lower blood sugar levels [11-13]. *G. lucidum* can also reduce liver damage caused by chemicals and oxidative stress [14]. However, the hepatoprotective effect of polysaccharides extracted from *G. lucidum* against liver damage in animal models remains unclear. Silymarin is a polyphenolic flavonoid isolated from milk thistle (*Silybum marianum*) which has a history as a medical plant for almost two millennia. Some studies indicate that silymarin shows strong antioxidant activity, and it is used clinically in Europe and Asia for the treatment of liver disease [15,16]. In this study, silymarin was used as a positive control.

This study aimed to explore the hepatoprotective effect of crude polysaccharides extracted from *G. lucidum* against liver damage in CCl_4_-treated mice.

## Materials and Methods

### Ethical approval

Ethical approval for this study was obtained from the Committee of Animal Care and Use, Faculty of Veterinary Medicine, Universitas Airlangga, Surabaya, Indonesia (approval no. 2.KE.168.10.2018).

### Materials and chemicals

*G. lucidum* basidiocarps were obtained from Tulungagung, East Java, Indonesia. Taxonomic identification of *G. lucidum* was carried out by Dr. Ni’matuzahroh from the Department of Biology, Faculty of Science and Technology, Airlangga University, Surabaya, Indonesia. Alanine aminotransferase (ALT), aspartate aminotransferase (AST), malondialdehyde (MDA), superoxide dismutase (SOD), and catalase (CAT) enzyme-linked immunosorbent assay (ELISA) kits were purchased from Bioassay Technology Laboratory (Shanghai, China). All other chemicals and solvents used were of analytical grade.

### Preparation of crude polysaccharides from *G. lucidum*

Dried *G. lucidum* powder was extracted with water at 90-100°C for 6 h. The solution was centrifuged at 4300× g for 5 min. The whole extract was filtered, concentrated, and centrifuged, and the supernatant was precipitated with absolute ethanol (3× volume of supernatant) at 4°C overnight. The resulting precipitate was washed with 70% ethanol and dried under vacuum at 40°C to obtain the crude polysaccharides.

### Animals

Male BALB/C mice with a bodyweight (bw) of 25±2 g were obtained from the Faculty of Pharmacy, Universitas Airlangga (Surabaya, Indonesia). They were maintained under a controlled temperature of 25±2°C, humidity of 50±10%, and a 12 h light-dark cycle and had free access to food and water.

### Experimental design

Mice were randomly divided into six groups (n=4/each). Group I (the normal control) was orally administered 0.5% carboxymethyl cellulose (CMC) once a day for 8 days and was administered an intraperitoneal injection of olive oil on day 9; Group II (the silymarin group) was orally administered 50 mg/kg bw silymarin daily for 8 days and was administered an intraperitoneal injection of CCl_4_ on day 9; Group III (the CCl_4_ group) was orally administered 0.5% CMC daily for 8 days and was administered an intraperitoneal injection of CCl_4_ on day 9; Group IV (the low *G. lucidum* group) was orally administered 50 mg/kg bw *G. lucidum* daily for 8 days and was administered an intraperitoneal injection of CCl_4_ on day 9; Group V (the medium *G. lucidum* group) was orally administered 100 mg/kg bw *G. lucidum* daily for 8 days and was administered an intraperitoneal injection of CCl_4_ on day 9; and Group VI (the high *G. lucidum* group) was orally administered 200 mg/kg bw *G. lucidum* daily for 8 days and was administered an intraperitoneal injection of CCl_4_ on day 9. The *G. lucidum* extract and silymarin suspension were suspended in 0.5% CMC. CCl_4_ was dissolved in olive oil (1% V/V, 5 mL/kg). On day 10, mice were anesthetized with ketamine/xylazine and sacrificed by cervical dislocation. Serum samples were collected in tubes. Liver samples were quickly excised and washed immediately in phosphate-buffered saline (PBS) to remove blood. One part of each liver sample was immediately stored at −20°C until analysis, and the other was fixed in 10% formalin solution for histopathologic analysis.

### Measurement of serum AST and ALT levels

Blood samples were collected in tubes and then centrifuged at 3000× g for 20 min. To assess liver damage, serum AST and ALT levels were assayed using ELISA kits.

### Measurement of hepatic MDA, SOD, and CAT levels

Liver samples were homogenized in PBS (3 mL) to produce liver homogenates. The homogenates were then centrifuged at 3000× g for 20 min at 4°C. The levels of MDA, SOD, and CAT in the supernatants were then measured using ELISA kits, according to the manufacturer’s instructions.

### Histological examination

The fresh liver tissues were trimmed into 3-mm thick slices, placed in cassettes, and immersed in neutral buffered formalin for 24 h. The fixed tissues were embedded in paraffin, sectioned, deparaffinized, and rehydrated using standard techniques. To examine histological changes, the liver sections were stained with hematoxylin and eosin and were subsequently examined under a light microscope (OLYMPUS CX23) at 40×.

### Statistical analysis

Data are expressed as mean±standard deviation. Differences between the groups were analyzed using one-way analysis of variance followed by Duncan’s *post hoc* test using SPSS version 21 (SPSS Inc., Chicago, IL, USA). p<0.05 was considered statistically significant.

## Results

### Effect of *G. lucidum* on serum ALT and AST levels

The CCl_4_-treated mice exhibited significantly increased levels of ALT and AST compared to the control group. However, *G. lucidum* treatment for 8 days significantly decreased the levels of ALT and AST (Table-1).

**Table-1:** Effect of *G. lucidum* extract on liver marker enzymes in mice treated with carbon tetrachloride.

Group	ALT	AST
Normal control	8.351±2.35	12.301±4.3
Silymarin	6.718±1.356	16.234±0.734
Carbon tetrachloride	16.180±4.158***	29.644±4.009***
Low *G. lucidum*	5.777±0.339**	11.279±0.195
Medium *G. lucidum*	5.637±0.517**	11.224±0.503
High *G. lucidum*	5.711±0.665	10.680±1.1

Data are expressed as mean±standard deviation (n=6).

** p<0.05 compared to the normal control group.

*** p<0.05 compared to all groups. ALT=Alanine transaminase, AST=Aspartate transaminase, *G. lucidum=Ganoderma lucidum*

### Effect of *G. lucidum* on hepatic MDA, SOD, and CAT levels

The CCl_4_-treated mice exhibited significantly increased hepatic MDA levels and significantly decreased SOD and CAT levels compared to the normal control group. The administration of *G. lucidum* extract attenuated these CCl_4_-induced alterations in MDA, SOD, and CAT levels (Table-2).

**Table-2:** Effect of *G. lucidum* extract on MDA, SOD, and CAT levels in mice treated with carbon tetrachloride.

Group	MDA	SOD	CAT
Normal control	1.246±0.126	5.945±1.145	2.852±0.716
Silymarin	1.543±0.152**	6.057±1.082	3.119±0.366
Carbon tetrachloride	2.858±0.303***	4.621±0.207***	2.312±0.080***
Low *G. lucidum*	1.952±0.329**	5.671±0.525	2.797±0.360
Medium *G. lucidum*	1.892±0.146**	6.236±0.869	3.009±0.466
High *G. lucidum*	1.510±0.219	6.246±0.814	3.178±0.368

Data are expressed as mean±standard deviation (n=6).

** p<0.05 compared to the normal control group.

*** p<0.05 compared to all groups. MDA=Malondialdehyde, SOD=Superoxide dismutase, CAT=Catalase, *G. lucidum*=*Ganoderma lucidum*

### Effect of *G. lucidum* on histological changes in the liver

**Figure-1:**
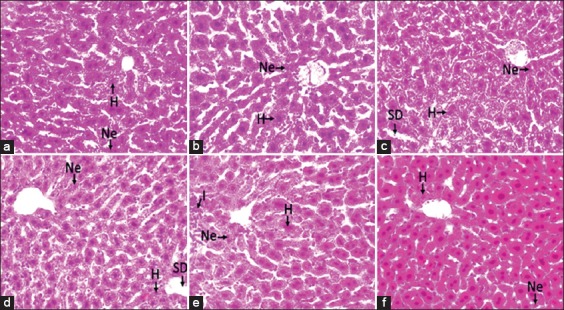
Effect of *Ganoderma lucidum* extract on liver histology in mice. N=Normal cell, Ne=Necrotic cell, H=Hydropic cell, SD=Sinusoidal dilatation, I=Inflammation. (a) Normal control group; (b) silymarin group; (c) carbon tetrachloride group; (d) low *G. lucidum* (50 mg/kg body weight [bw]) group; (e) medium *G. lucidum* (100 mg/kg bw) group; (f) high *G. lucidum* (200 mg/kg bw) group.

The liver tissue of the normal control group exhibited normal liver architecture such as distinct hepatocytes, sinusoidal spaces, and a clear central vein. The liver tissue of the CCl_4_-administered silymarin group showed only mild hydropic degeneration and a low number of necrotic cells. The liver tissue of the CCl_4_-treated group showed a high number of necrotic cells, hydropic degeneration, and sinusoidal dilatation. The liver tissue of the group administered a 50 mg/kg bw dose of *G. lucidum* extract showed sinusoidal dilatation and moderate hydropic degeneration and necrosis. The liver tissue of the group administered a 100 mg/kg bw dose of *G. lucidum* extract showed reduced hydropic degeneration and fewer necrotic and inflammatory cells. The liver tissue of the group administered a 200 mg/kg bw dose of *G. lucidum* extract showed very little hydropic degeneration and necrosis (Figure-1).

## Discussion

Recently, natural products have become widely used for the treatment of liver disease. This study aimed to investigate the hepatoprotective effect of crude polysaccharides extracted from *G. lucidum* against liver damage in mice. Polysaccharides, found in animals, plants, and other microorganisms, have antioxidant and anti-inflammatory potential and can protect the liver from damage caused by exposure to chemicals [17,18]. *G. lucidum* has been reported to have antitumor, cardioprotective, antioxidant, antibacterial, and antiviral effects [19-21]. In the previous studies, *G. lucidum* extract prevented damage caused by diseases associated with ROS [22-24]. Furthermore, the protective effect of *G. lucidum* extract against oxidative stress-induced liver damage has been investigated. In this study, the protective effect of polysaccharides extracted from *G. lucidum* against CCl_4_-induced hepatotoxicity was investigated.

CCl_4_-induced liver damage is commonly used to experimentally study the hepatoprotective effects of drugs [25,26]. Cytochrome P450 metabolizes CCl_4_ to trichloromethyl radicals (CCl_3_^-^) in the endoplasmic reticulum of liver cells, and these bind to O_2_ to form trichloromethyl peroxyl(CCl_3_OO^-^) radicals. These radicals subsequently bind to cell molecules such as unsaturated fatty acids, ultimately causing lipid peroxidation. Furthermore, the structure and function of the cell membrane become disrupted. In this study, CCl_4_ administration induced acute damage to liver cells, demonstrated by increased serum ALT and AST levels. ALT and AST are often considered sensitive markers for diagnosing liver damage due to their presence of the cytoplasm facilitates blood flow after liver cell damage [27]. In this study, *G. lucidum* extract significantly reduced ALT and AST levels, demonstrating that it can prevent cell damage.

The histological results obtained correlated with the aforementioned biochemical results. Histological signs of liver damage include hydropic degeneration, inflammation, and necrosis. The increasing permeability of the liver cell membrane causes metabolic disorders, inhibits protein synthesis, and produces necrosis-inducing cellular degradative enzymes. The increased number of inflammatory cells in damaged liver cells causes an increase in ROS levels. Hydropic cells are formed when the membrane cell transport system fails, resulting in excessive water in the cell. The liver tissue of the mice administered *G. lucidum* showed histopathological improvements, indicating that *G. lucidum* can protect against CCl_4_-induced liver damage.

Lipid peroxidation in the liver cell membrane is an important oxidative stress parameter. In addition, lipid peroxidation is also caused by free radicals derived from CCl_4_ [28]. The increased levels of MDA noted after CCl_4_ administration indicate the occurrence of lipid peroxidation. This demonstrates the occurrence of liver cell damage and the failure of antioxidants to prevent abundant free radicals [29]. In this study, the administration of *G. lucidum* significantly reduced MDA levels. This extract can act as a free radical scavenger and can protect membrane lipids from oxidative damage. Cell protection against free radical attacks is dependent on radical scavengers such as SOD and CAT. SOD converts superoxide radicals to H_2_O_2_, and this is then metabolized by CAT into H_2_O and O_2_, ultimately preventing liver cell damage caused by these free radicals [30]. SOD and CAT levels increased in the mice administered *G. lucidum*, demonstrating that *G. lucidum* extract can restore and maintain the activity of SOD and CAT. The previous studies have suggested that CCl_4_ reduces the activity of antioxidant enzymes and causes hepatopathy [31,32]. The administration of *G. lucidum* can thus protect against free radicals, protecting hepatocytes from hepatopathy.

## Conclusion

In this study, *G. lucidum* administration decreased the levels of ALT, AST, and MDA and increased the levels of SOD and CAT in CCl_4_-treated mice. This indicates that *G. lucidum* has significant hepatoprotective activity, which likely results from its antioxidant activity. Ultimately, *G. lucidum* could be developed as a novel protective agent against acute liver damage.

## Authors’ Contributions

WD has made a significant contribution to conception, design, interpretation of data, drafting, revising the manuscript, and gave final approval of the version to be published. RJKS made a substantial contribution to the acquisition of data, analysis, and drafting of the manuscript. DW designed the study, analyzed data, drafted the article, and made critical revision. SAH helped in collection data, edited article, and made critical revisions. SH analyzed data and draft articled. HP performed drafted the article and made critical revision. SPAW edited the article and made critical revisions. ESS participated in collection data. All authors read and approved the final manuscript.
